# Pluripotent Stem Cell-Derived Mesenchymal Stem Cells Show Comparable Functionality to Their Autologous Origin

**DOI:** 10.3390/cells10010033

**Published:** 2020-12-28

**Authors:** Mark Jakob, Mario Hambrecht, Jennifer L. Spiegel, Julia Kitz, Martin Canis, Ralf Dressel, Katrin Streckfuss-Bömeke

**Affiliations:** 1 Department of Otorhinolaryngology, LMU Klinikum, Ludwig-Maximilians-Universitaet Muenchen, 81377 Munich, Germany; jennifer.spiegel@med.uni-muenchen.de (J.L.S.); martin.canis@med.uni-muenchen.de (M.C.); 2 Department of Otorhinolaryngology, University Medical Center Goettingen, 37075 Goettingen, Germany; mario.hambrecht@stud.uni-goettingen.de; 3 Institute of Pathology, Medical Center Goettingen, 37075 Goettingen, Germany; j.kitz@med.uni-goettingen.de; 4 Institute of Cellular and Molecular Immunology, University Medical Center Goettingen, 37075 Goettingen, Germany; rdresse@gwdg.de; 5 Clinic for Cardiology and Pneumology, University Medical Center Goettingen, 37075 Goettingen, Germany

**Keywords:** pluripotent stem cell-derived mesenchymal stem cells, iP-MSCs, head and neck

## Abstract

A multimodal therapeutic approach involving radiotherapy is required when treating head and neck squamous cell carcinoma. However, radiotherapy is restricted due to its high risk for damages to the surrounding healthy tissue of the treated area. Tissue regeneration and wound healing is promoted by the survival and regenerative capacities of tissue-resident or invading stem cells. Mesenchymal stem cells (MSCs) exhibit a promising therapeutic potential in the field of cell-based tissue engineering and regenerative medicine due to their immunomodulatory properties and differentiation capacity. However, the generation of MSCs for therapeutic applications is still a major challenge. We aimed to produce highly homogeneous induced pluripotent stem cell-derived mesenchymal stem cells (iP-MSCs) in an autologous manner from initially isolated human mucosa mesenchymal stem cells (mMSCs) of the upper respiratory tract. Therefore, mMSCs were reprogrammed into induced pluripotent stem cells (iPSCs) by non-integrative chromosomal technologies and differentiated into corresponding iP-MSCs. We demonstrated that mMSCs and iP-MSCs show similar cell characteristics in terms of morphology, clonogenic potential, differentiation, and surface phenotype. Moreover, iP-MSCs demonstrated related immunosuppressive capacity as mMSCs including the secretion of cytokines, and T cell inhibition. Therefore, generating iP-MSCs in an autologous manner may be a novel personalized treatment option in regenerative medicine.

## 1. Introduction

Treatment of head and neck squamous cell carcinoma is based on a multimodal therapeutic approach involving the resection of the tumor, adjuvant radiotherapy, and chemotherapy [1]. The efficacy of radiation therapy is associated with severe adverse effects, including mucositis, pain, and xerostomia, mainly due to the damages of the surrounding healthy tissue of the treated area. Promotion of tissue regeneration and wound healing is crucial and depends primarily on the survival and regenerative properties of tissue-resident or invading stem cells [2,3]. Mesenchymal stem cells (MSCs) exhibit a promising therapeutic potential in the field of cell-based, tissue-engineering, and regenerative medicine due to their immunomodulatory properties and differentiation capacity [4]. They are multipotent, non-hematopoietic, plastic adherent fibroblast-like progenitor cells and characterized by certain cell surface markers (CD29, CD44, CD105, CD73, CD90, and CD106) [5,6]. In addition, they have the capability to differentiate into multiple cell lineages including chondrocytes, osteoblasts, adipocytes, tenocytes, or myocytes [7,8].

Moreover, MSCs are described to exhibit immunosuppressive properties due to immunomodulation of dendritic cells, T cells, B cells, and natural killer (NK) cells [9,10,11,12]. They have been shown to promote tissue repair and regeneration in vitro and hold promise for MSC-based therapies of tissue damage, including ionized radiation-induced lesions [13,14]. MSCs of the head and neck region have been found to promote the regenerative function of NK cells, which in turn mediate angiogenesis and tissue growth in peripheral tissues to initiate repair after infection and inflammation [15]. Furthermore, systemic or local application of MSCs results in a remarkable therapeutic benefit in graft-versus-host disease, Crohn’s disease, and chronic wounds, such as diabetic ulcers [6]. In cases of xerostomia, patients reported a significant reduction of symptoms after local injection of adipogenous MSCs within a placebo-controlled randomized trial [16].

However, a functional heterogeneity within MSCs is reported, which might be based on differences in donor source, culture protocols, and expansion levels [17]. MSCs derived from different tissues (e.g., bone marrow, fat, mucosa, or parotid gland) exhibit diverse biological properties [18,19,20]. Consequently, the extension of homogeneous MSCs bears a major challenge in order to meet specific criteria for therapeutic applications. Under radiation, MSCs from the head and neck area retain their MSC-like capacities [21]. The interaction characteristics with other immune cells via cytokines and chemokines appear maintained, as well as the migration potential. However, post-radiation, the potential of replication is impaired, which could possibly lead to a depletion of the local MSC-pool after irradiation.

The generation of human-induced pluripotent stem cells (iPSCs) from adult somatic cell sources opens the possibility to generate high numbers of cell populations on a patient-specific level [22]. iPSC-derivatives have been widely used to study hereditary diseases *in vitro* with a correlation to the predicted phenotypes [23,24,25]. In addition, iPSC-derivatives were used for transplantation in order to enhance regenerative capacity in the injured tissue [26]. However, they could represent an important alternative source for functional MSCs. MSCs are well-established somatic cell sources for reprogramming into iPSCs with high reprogramming efficiencies [22,27]. Vice versa, iPSCs have been successfully differentiated into functional MSCs (iP-MSCs) with high efficiency [28], which might potentially serve as an ideal source for homogeneous and high-quality MSCs. However, less is known about the functional and biological similarities of primary isolated human MSCs and generated autologous iP-MSCs.

**Figure 1:**
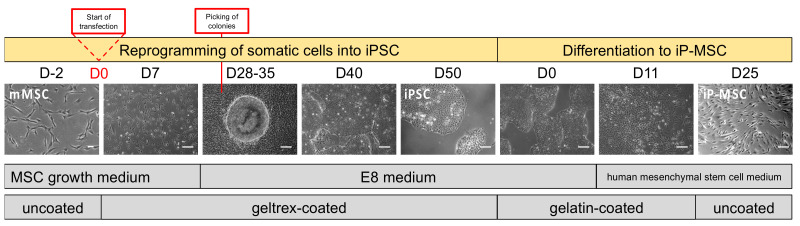
Time scale of reprogramming from nasal mucosal mesenchymal stem cells (mMSCs) to induced pluripotent stem cells (iPSCs) and differentiation towards induced pluripotent stem-cell-derived mesenchymal stem cells (iP-MSCs). The mMSCs are seeded on day (D)2 in MSC growth medium (scale bar 100 μm). On D0, mMSCs were transfected using episomal plasmids for reprogramming. First, morphology changes were visible on D7 followed by medium change to E8 (scale bar 100 μm). iPSC colonies appeared between D28 and D35 after transfection (scale bar 100 μm). Potential iPSC colonies were mechanically picked and maintained in culture for further passages, reaching typical pluripotent stem cell morphology around D50 (scale bar 100 μm). Differentiation of iPSCs into MSCs started with high-density iPSCs on gelatin-coated plates (scale bar 100 μm). E8 medium was changed to human mesenchymal stem cell medium at D11 after initiation of differentiation (scale bar 100 μm). Seven days later, the cells were transferred to uncoated plates, and the first mesenchymal-like cells appeared on D25 after starting differentiation (scale bar 100 μm).

In the present study, we show that human mucosa MSCs (mMSCs) of the upper respiratory tract were isolated and cultivated, reprogrammed into iPSCs and differentiated into corresponding highly homogeneous induced pluripotent MSCs (iP-MSCs) (Figure 1). We investigated similarities and differences in cell characteristics (morphology, clonogenic potential, differentiation, and cell surface phenotype), as well as the immunomodulatory potential (secretion of cytokines, and T cell inhibition) of primary isolated mMSCs and artificially generated iP-MSCs.

## 2. Materials and Methods

### 2.1. Isolation and Culture of Human Adult MSC 

Isolation of human tissue-resident MSC from nasal mucosa (mMSC) was performed from 4 patients (median age 40 years; range 21 to 77 years, Table 1). The study and all experiments were conducted in full accordance with ethical principles, including the World Medical Association Declaration of Helsinki (version 2002) and additional requirements. Prior to the experiments, all patients gave informed consent to the study approved by the ethics committee of the Universitätsmedizin Göttingen, Georg-August-Universität Göttingen, Göttingen, Germany, with ethics number 3/4/17 and 10/9/15. As described before, tissue was retrieved from healthy individuals undergoing a conchotomy of the lower turbinate [21,29]. Samples of the inferior nasal concha (<1 g) were transferred to 10 mL of a 0.9% sodium chloride (NaCl) solution after tissue resection, and mMSCs were isolated according to the isolation protocol described earlier [30].

Cell cultures were performed in MSC growth medium (94% Dulbecco’s Modified Eagle’s Medium-DMEM (Thermo Fischer Scientific, Waltham, MA, USA), 5% human platelet lysate (PL BioScience GmbH, Aachen, Germany), 1% penicillin/streptomycin (Thermo Fischer Scientific), and 0.04% heparin (Biochrom, Berlin, Germany)) at 37 °C and 5% CO_2_.

### 2.2. Generation and Culture of iPSCs

For plasmid-based integration-free reprogramming, the protocol was described earlier [31]. Briefly, 5 × 10^5^ mMSCs were used for electroporation with the NHDF Nucleofector Kit (Lonza, Basel, Switzerland): cells were suspended in nucleofection β solution and 1–2 µg of the plasmids pCXLE-hSK, pCXLE-hUL and pCXLEhOct3/4-shp53-F were used for each experiment. Electroporation was done with the nucleofector II (Lonza) with the program P22 or U23. Cells were plated on 6-well plates in MSC growth medium with 5 µmol/L pro survival factor (Millipore, Darmstadt, Germany) and 500 µmol/L sodium butyrate (Gibco Invitrogen, Karlsruhe, Germany). Medium was changed every other day with MSC growth medium with 500 µmol/L sodium butyrate. On day 7 post-transfection, transfected cells were harvested and plated on a Geltrex-coated 6-well plate for picking iPS-like colonies. About 3 to 4 weeks after the specific reprogramming procedure, colonies with iPS-like morphology were picked mechanically and gave rise to the different stem cell lines. Newly established stem cell lines were passaged with Versene solution (Thermo Fisher Scientific) and cultivated in E8 medium (Thermo Fisher Scientific) five passages before being used for experiments. The four iPSC lines were named iPSC2, iPSC3, iPSC5, and iPSC7. Two independent cell lines were generated per patient and used for each experiment.

### 2.3. Differentiation of iPSCs into iP-MSCs

iPSC-cultures with a confluency of about 80% received mesenchymal stem cell growth medium 2 (PromoCell, Heidelberg, Germany). The cells were transferred in a dilution of 1:2 to Geltrex-coated 6-well plates on day 7 with versene (Thermo Fisher Scientific) and on day 14 with accutase (Gibco Invitrogen). Cells were cultivated for 3 weeks and passaged at a high confluency of 90% and diluted 1:2–1:4 on uncoated plates. The time scale is depicted in Figure 1.

### 2.4. Flow Cytometry

A total of 5 × 10^4^–1 × 10^5^ cells (passage 4–6) were washed in phosphate-buffered saline (PBS), resuspended in 100 µL FACS buffer (PBS, 2% fetal calf serum), and incubated with the appropriate antibody or isotype control for 20 min at room temperature (RT) in the dark. Cells were washed in PBS and resuspended in 250 µL FACS buffer, and flow cytometry was performed using the flow cytometer FACS Canto II (Becton Dickinson Bioscience, Franklin Lakes, NJ, USA). Details regarding the used antibodies are listed in Table S1.

### 2.5. Mesodermal Differentiation

As described before, mMSCs were differentiated into adipogenic, osteogenic, and chondrogenic cells [21]. In brief, adipogenic and osteogenic differentiation was induced with mesenchymal stem cell adipogenic differentiation medium 2 (PromoCell) and osteogenic differentiation medium (PromoCell), respectively, by culturing 5 × 10^4^ cells on round glass slides in 6-well plates. After 14 days, the cells were fixed and stained with oil red O (adipogenic differentiation) and alizarine red (osteogenic differentiation) [21]. Chondrogenic differentiation was induced using micro mass body cultivation with chondrogenic differentiation medium. After centrifugation and incubation, mesenchymal stem cell chondrogenic differentiation medium (PromoCell) was added at 48 h and replaced every 3 days with fresh medium. After 21 days, the pellet was collected and put in paraffin wax, cut, and stained with Alcian blue.

### 2.6. Immunofluorescence Staining

Prior to immunofluorescence staining of cultured mMSCs, iPSCs, and iP-MSCs, 8 × 10^3^ cells were plated on 12 mm coverslips (the iPSCs on Geltrex) in 12-well plates (Greiner Bio-One, Kremsmuenster, Austria). The cells were washed with PBS, fixed with 4% PFA for 20 min at RT, and blocked with 0.1% bovine serum albumin (BSA) (Sigma-Aldrich, St. Louis, MO, USA) at 4 °C overnight. The primary antibodies were diluted with 1% BSA. For all antibodies against nuclear proteins such as OCT4, SOX2, LIN28, and NANOG, 0.1% Triton X-100 (Sigma-Aldrich) in PBS was added for permeabilization. It was disposed for 1 h at 37 °C or overnight at 4 °C. The secondary antibodies were diluted in 1% BSA and disposed for 1 h at RT. Nuclei were counter-stained with 4,6-diamino-2-phenylindole (DAPI, 0.2 ng/mL, Sigma-Aldrich) for 10 min at RT. For mountingof the samples, Fluromount-G (eBioscience, San Diego, MO, USA) was used. Details regarding the appropriated antibodies are listed in Table S2. The cells were examined with the Axio Observer.Z1 inverted microscope (Carl Zeiss, Göttingen, Germany). Detection of the FITC fluorophore was carried out by using the filter sets with excitation BP 475/40, beam splitter FT 500, and emission BP 530/50. Detection of the Cy3 fluorophore was done by using the filter sets with excitation BP 540/25, beam splitter FT 565, and emission BP 605/55. The filter sets with excitation G 365, beam splitter FT 395, and emission BP 445/50 were performed for the detection of the DAPI fluorophore.

### 2.7. Alkaline Phosphatase (ALP) Staining

Activity of Alkaline Phosphatase (ALP) in pluripotent cells was detected using the ALP Staining Kit from Sigma-Aldrich according to manufacturer’s protocol. Therefore, iPSC-colonies (6 cm dishes at 60% confluence) were washed with PBS, fixed, stained, and analyzed by light microscopy.

### 2.8. Embryoid Body Formation

For analyzing the spontaneous differentiation capacity of the iPSCs, an embryoid body (EB) formation was initialized. For this purpose, iPSCs were cultivated on mouse embryonic fibroblasts in hES-medium (DMEM/F12 (Thermo Fisher Scientific), 1% NEAA (Thermo Fisher Scientific), 1% β-mercaptoethanol (Serva Electrophoresis, Heidelberg, Germany), 15% knockout serum (Thermo Fisher Scientific). When the iPSCs had reached a confluency of 90–100%, cells were treated for 4 min with collagenase IV (200 U/mL) at 37 °C and scrapped into big clusters. The clusters were gently resuspended in hES-medium with a glass pipette and transferred to an uncoated 6 cm dish so that EBs formation could occur in suspension (day 0). After 24 h, medium was changed to Iscove-Differentiation Medium (Iscove medium; Thermo Fisher Scientific), 20% fetal calf serum (Sigma-Aldrich, St. Louis, MO, USA), 1% NEAA (Thermo Fisher Scientific), and monothioglycerol (450 µmol/L; Sigma-Aldrich) and changed every two days. After 8 days of cultivation, accrued EBs were resuspended onto Geltrex coated coverslips and fixed at day 8 + 5 (AFP) and day 8 + 25 (smooth muscle-α -actin (SM-α-actin), βIII-tubulin). RNA samples were taken and analyzed at day 0, 8, and 8 + 25.

### 2.9. Immunohistochemical Staining

Immunohistochemical staining for vimentin was performed with the autostainer (Vimentin Flex monoclonal, mouse V9, autostainer Dako, IR63061-2; Agilent, Santa Clara, CA, USA). The cells were washed with buffer solution, pre-treated with an enzyme, and washed again with buffer solution. Then, an endogenous enzyme bloc (flex peroxidase bloc) was applied for 5 min and the cells were washed again with buffer solution. After adding the first antibody, the cells were washed again, and the labeled polymer (flex/HRP) was added for 20 min. Another two rounds of washing the cells with a buffer were followed by a 10-min period of incubation with the substrate chromogen (Flex DAB + sub-chromo). Then, the cells were washed with buffer solution again, counterstained with flex hematoxylin, washed with the buffer, and finally washed with distilled water.

### 2.10. Reverse Transcriptase-Polymerase Chain Reaction (RT-PCR)

For total RNA isolation, the SV Total RNA Isolation System with on-column DNase digestion (Promega, Madison, WI, USA) was used. The first-strand cDNA synthesis was carried out with DNase-treated RNA (200 ng) by using Murine Leukaemia Virus Reverse Transcriptase and Oligo d(T)16 (Applied Biosystems, Forster City, CA, USA). One-tenth of the cDNA reaction was taken as PCR template and amplified for 25–35 cycles depending on the relative mRNA quantity with denaturation at 94 °C for 15 s, annealing at 58 °C to 64 °C for 15 s according to the primers, and elongation at 72 °C for 30 s. The forward and reverse primer sequences, the annealing temperatures, and the number of cycles being performed for RT-PCR analyses are listed in Table S3. GAPDH was used as an internal control.

### 2.11. Enzyme-Linked Immuno-Sorbent Assay (ELISA)

IL-6 and IL-8 concentrations in the supernatants of mMSCs, iPSCs, and iP-MSCs were determined after culturing 5 × 10^5^ cells in wells of 12-well plates in 1 mL medium for 24 h by using ELISA sets for human IL-6 (BioLegend, San Diego, CA, USA) and IL-8 (BioLegend) according to the manufacturer’s instructions. The samples were measured using a BioTek PowerWave 340 microplate spectrophotometer (BioTek, Bad Friedrichshall, Germany) set to 405 nm.

### 2.12. Inhibition of T Cell Proliferation

Peripheral blood mononuclear cells (PBMCs) were obtained from healthy blood donors by centrifugation on Biocoll separating solution (Biochrom). CD4^+^ T cells were isolated from PBMCs by magnetic-activated cell sorting using a negative selection kit (CD4^+^ T cell isolation kit, 130-096-533; Miltenyi Biotec, Bergisch-Gladbach, Germany). The purified CD4^+^ T cells were labeled by incubation for 5 min with 5 µM carboxyfluorescein succinimidyl ester (CFSE) (C-1157, Gibco Invitrogen) in phosphate-buffered saline at 37 °C. After being washed 3 times with DMEM containing 10% fetal calf serum (FCS), the cells were added to plates prepared for stimulation of T cell proliferation. For this purpose, 96-well Nunc MaxiSorp microtiter plates were coated overnight at 4 °C with 10 µg/mL goat anti-mouse IgG F(ab’) 2 fragments in sodium carbonate buffer (pH 8.5; 100 µL/well). After washing with PBS, stimulatory anti-CD3 (UCHT1, low endotoxin acid-free-LEAF, mouse IgG_1_; BioLegend) and anti-CD28 antibodies (CD28.2, LEAF, mouse IgG_1_, BioLegend) were added at concentrations of 0.01 µg/mL (anti-CD3) and 0.5 µg/mL (anti-CD28) and incubated at room temperature for 2 h. Then the plates were washed again with PBS and incubated for 1 h at 37 °C with 0.1% gelatine/PBS before 1 × 10^5^ freshly isolated CD4^+^ T cells per well were added in 100 µL medium with 10% FCS. The CD4^+^ T cells were cultured either alone (0:1) or in presence of different ratios of mMSCs, iPSCs, or iP-MSCs (0.5:1, 1.1, or 2:1) for 4 days. Then, the cells were harvested and stained by an anti-CD4 antibody (RPA-T4, mouse IgG_1_, PE/Cy3-labeled, BioLegend). Flow cytometry was performed as described previously [32] on a BD LSRII flow cytometer (BD Biosciences, Heidelberg, Germany) using FlowJo software (TreeStar, Ashland, Wilmington, NC, USA) for data analysis. The proportion of proliferating CD4^+^ T cells with reduced CFSE fluorescence intensity was determined after gating of lymphocytes in forward/side scatter plots and gating on CD4^+^ cells.

### 2.13. Statistical Analysis

Data are given as the mean ± standard error of the mean (SEM). The Kruskal–Wallis test or the two way-ANOVA was used for comparison of more than two groups and multiple comparisons. A value of *p* less than 0.05 was considered statistically significant. * = *p* ≤ 0.05, ** = *p* ≤ 0.01; *** = *p* ≤ 0.001. Statistical analysis was done with GraphPad prism 6–8. Numbers of analyzed samples are defined for each experiment in the figure legend.

## 3. Results

### 3.1. Isolated mMSCs From Nasal Mucosa Show Typical Tissue-Derived MSC Characteristics

**Figure 2:**
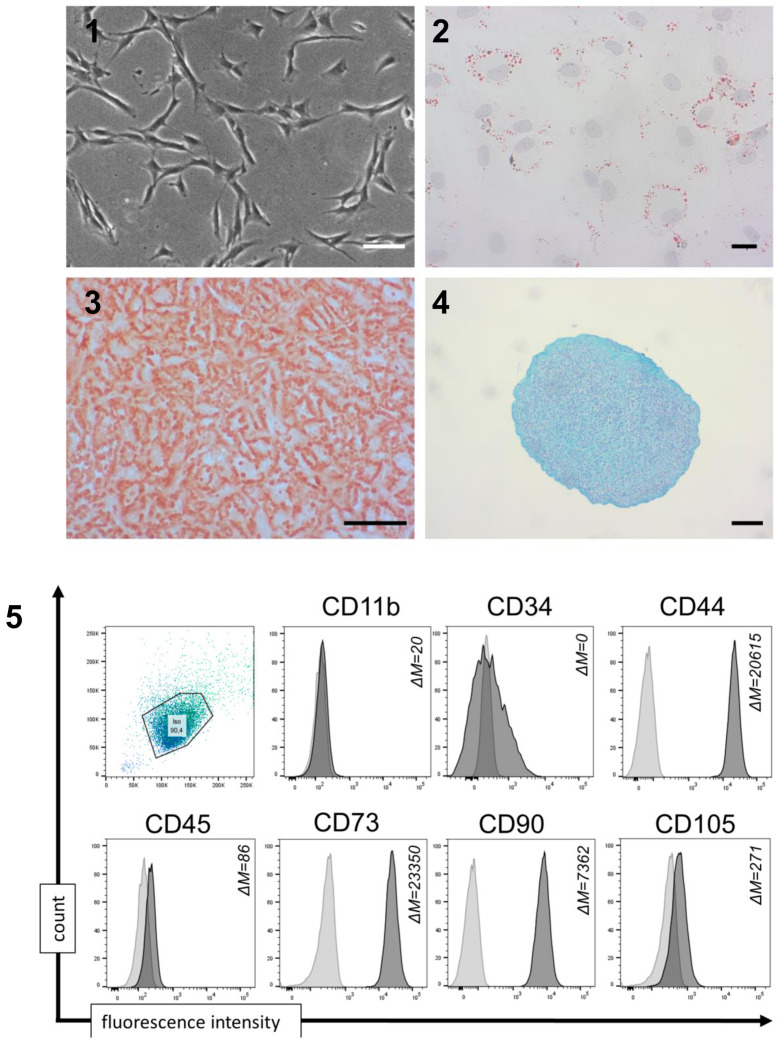
Characterization of nasal mucosa mesenchymal stem cells (mMSCs). (**1**) Representative images from mMSCs in passage 4 to 6 after isolation (mMSC3; scale bar 100 μm), (**2**–**4**) Representative images of immunohistochemistry staining of nasal mucosa MSCs for successful differentiation into adipocytes, osteocytes, and chondrocytes. (**2**) Adipogenic differentiation is detected with Oil Red O staining, (**3**) osteogenic differentiation is detected with Alizarin Red, and (**4**) chondrogenic differentiation is detected with Alcian Blue (scale bar 100 μm). (**5**) Representative diagrams of flow cytometric analyses of nasal mMSCs at passage 4-6 (n = 4) for cell surface markers being expressed (CD44, CD73, CD90, and CD105) and cell surface markers being absent (CD11b, CD34, and CD45). Data are shown as an overlay histogram with the isotype control (light gray) and cell surface marker staining (dark gray) (ΔM = mean fluorescence intensity, MFI; difference between MFI measure and isotype control).

mMSCs were isolated from the nasal mucosa, expanded, and characterized regarding morphological and cellular criteria. Tissue-derived mMSCs demonstrated characteristic mesenchymal spindle-shaped morphology and the ability to adhere to plastic (Figure 2 (1)). Most importantly, the isolated mMSCs featured the capacity to differentiate into the adipogenic, osteogenic, and chondrogenic cell lineages as shown by Oil Red O, Alizarin Red, and Alcian Blue staining (Figure 2 (2–4)). Flow cytometry analysis indicated a characteristic immunophenotypic profile with high expression of CD44, CD73, CD90, and CD105, while largely lacking CD11b, CD34, and CD45 (Figure 2 (5), Table S4).

### 3.2. mMSCs Can Be Efficiently Reprogrammed into iPSCs

**Figure 3:**
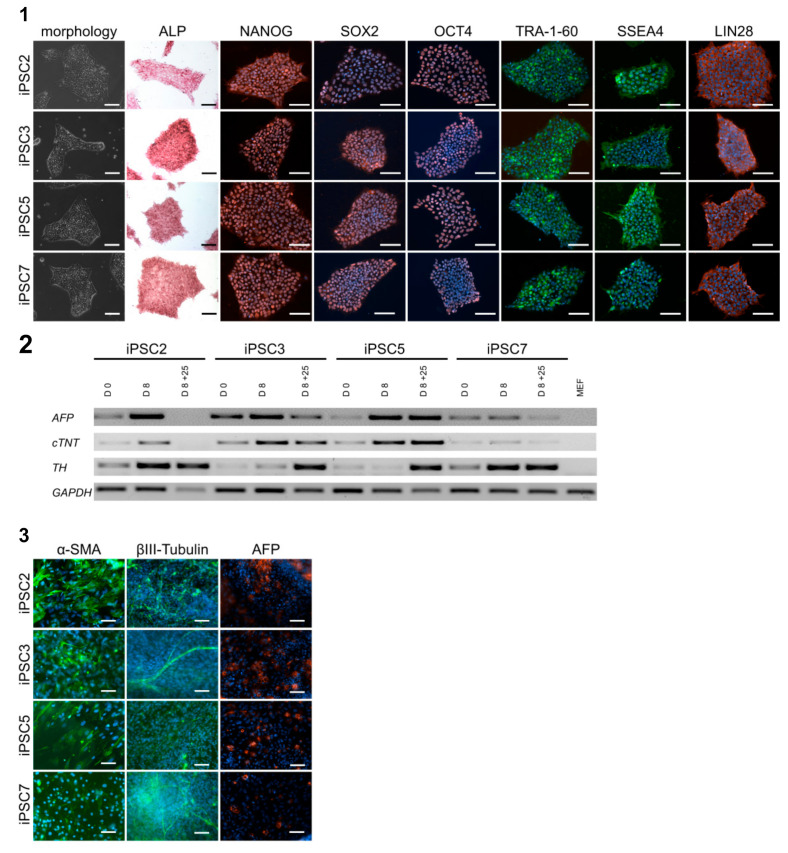
Characteristics of human induced pluripotent stem cells (iPSCs) derived from mucosa mesenchymal stem cells (mMSCs). (**1**) Generated iPSC-lines (iPSC2, 3, 5, 7) show typical morphology, are positive for alkaline phosphatase (ALP) activity, and show protein expression of pluripotency-related markers NANOG, SOX2, OCT4, TRA1-60, SSEA4, LIN28. Nuclei were stained with 4′,6-diamidino-2-phenylindole (DAPI) (scale bars 100 µm). (**2**–**3**) In vitro spontaneously differentiated iPSCs into embryoid bodies (EB) and analyzed for the presence of derivatives of all three germ layers. (**2**) Gene expression of endodermal α-fetoprotein (AFP), mesodermal cardiac troponin T (cTNT), and ectodermal tyrosine hydroxylase (TH) was upregulated during spontaneous differentiation (day 8, day 8 + day 25). GAPDH was used as a housekeeping gene, and mouse embryonic fibroblasts (MEFs) were used as negative control. (**3**) Immunostaining of EB outgrowths revealed the expression of endodermal AFP, mesodermal (α-SMA), and ectodermal (βIII-tubulin) marker proteins. Nuclei were stained with DAPI (scale bars 100µm).

The human tissue-derived mMSCs were reprogrammed into iPSCs by using the plasmid-based integration-free reprogramming method. Two independent cell lines per patient were analyzed for their pluripotency. The reprogrammed MSC-derived cells showed typical iPSC morphology and were positive for alkaline phosphatase (Figure 3 (1)). Each of the iPSC lines could have been maintained for 20 passages without any obvious phenotypic changes. No differences between iPSCs of younger or elder donors were observed. Immunocytochemical staining showed high expression of pluripotency markers NANOG, SOX2, OCT4, TRA-1-60, SSEA4, and LIN28 (Figure 3 (1)). One key feature of pluripotent stem cells is their spontaneous in vitro differentiation potential: Using the EB formation method, the iPSCs were shown to be able to differentiate in all three germ layers, which was confirmed in our experiments on mRNA and protein levels. EBs of all four patients were confirmed to express mRNA of tissue-specific germ layer markers. The early endodermal marker gene α-fetoprotein (AFP) and the mesodermal marker cardiac troponin T (cTNT) were already expressed on day 8, whereas the ectodermal marker tyrosine hydroxylase (TH) was expressed at later stages during the process of differentiation (Figure 3 (2)). The immunocytochemical staining resulted in high expression of the mesodermal marker α smooth muscle-actin (α-SMA), the ectodermal marker βIII-tubulin, and the endodermal marker AFP (Figure 3 (3)).

### 3.3. MSC-Derived iPSCs Can Be Differentiated into Functional iP-MSCs

**Figure 4:**
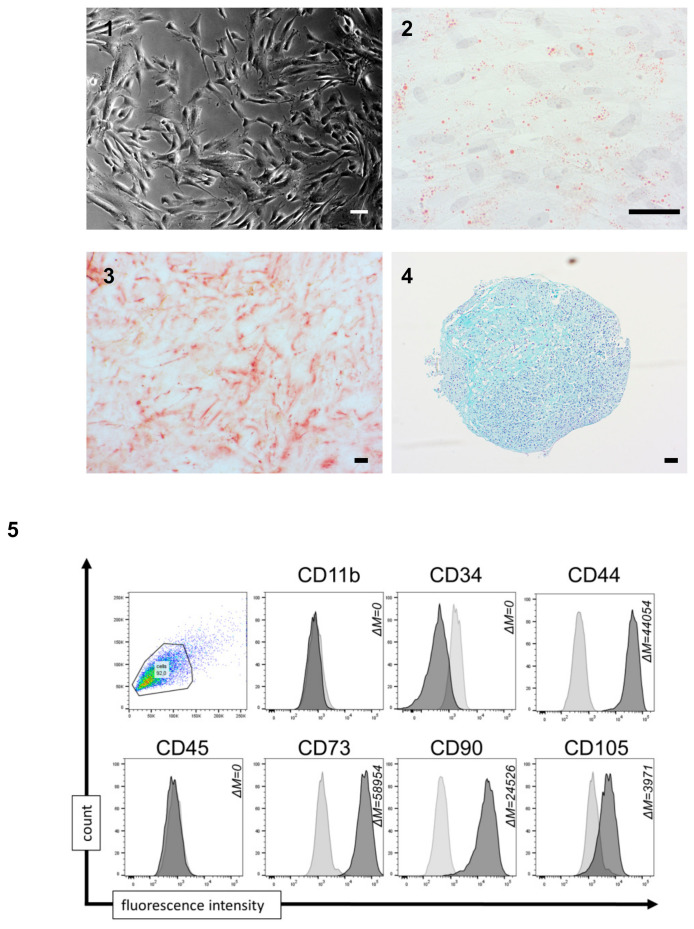
Characterization of induced pluripotent stem cell-derived mesenchymal stem cells (iP-MSCs). (**1**) Representative images from differentiated iP-MSCs in passage 3 (iP-MSC 3; scale bar 100 μm). (**2**–**4**) Representative images of immunohistochemistry staining of iP-MSCs showing successful differentiation into adipocytes, osteocytes, and chondrocytes. (**2**) Adipogenic differentiation is detected with Oil Red O staining. (**3**) Osteogenic differentiation is detected with Alizarin Red, and (**4**) chondrogenic differentiation is detected with Alcian Blue (scale bars 100 μm). (**5**) Representative diagrams of flow cytometric analyses of iP-MSCs at passage 3 (n = 4) for cell surface markers being expressed (CD44, CD73, CD90, and CD105) and cell surface markers being absent (CD11b, CD34, and CD45). Data are shown as an overlay histogram with the isotype control (light gray) and cell surface marker staining (dark gray) (ΔM = mean fluorescence intensity, MFI; difference between MFI measure and isotype control).

In order to differentiate the MSC-derived iPSCs into autologous MSCs (iP-MSCs), we used the gelatin-coated flat culture and mesenchymal stem cell growth medium 2 for the dilution after passaging the selected MSC precursor cells and separating the slow-growing differentiated cell types from the non-differentiated iPSCs. After several passagings (2–3), the mixed morphology disappeared and the proportion of cells with fibroblast-like morphology increased. After almost 4 weeks of culturing, the morphology was similar to regular mMSCs (Figure 4 (1)).

We characterized the iPSC-derived fibroblast-like cells regarding MSC properties. All iP-MSC lines had the capacity to differentiate into the three lineages of mesoderm origin including adipogenic differentiation shown by lipid droplets in the Oil Red O staining (Figure 4 (2)), osteogenic differentiation shown by histological staining of Alizarin red S (Figure 4 (3)), and chondrogenic differentiation with aggrecan-positive areas (Figure 4 (4)). Using flow cytometry, we characterized the iPSC-derived iP-MSCs regarding the expression of typical cell surface markers. The iP-MSCs were highly positive for CD44, CD73, CD90, and CD105, whereas CD11b, CD34 and CD45 were not expressed or less expressed by these cells (Figure 2 (5), Table S4). In conclusion, the generated iP-MSCs showed the functional and phenotypic characteristics of the original mMSCs isolated from the nasal mucosa.

### 3.4. Comparison of the Three Stem Cells Types

**Figure 5:**
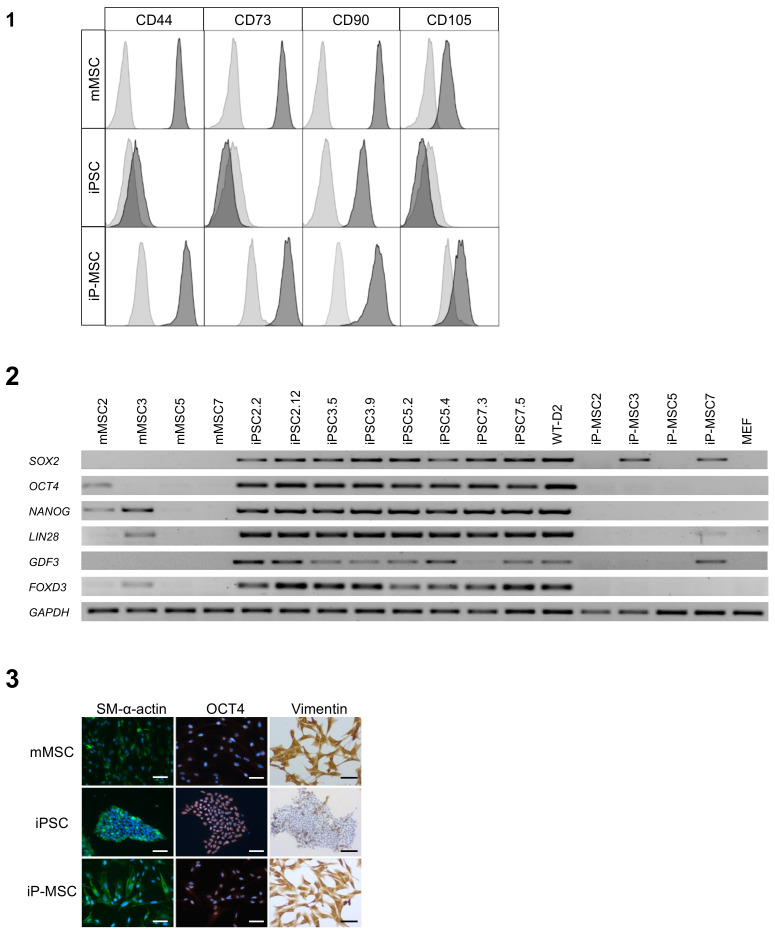
Comparison of nasal mucosa mesenchymal stem cells (mMSCs), induced pluripotent stem cells (iPSCs), and induced pluripotent stem cell-derived mesenchymal stem cells (iP-MSCs). (**1**) Flowcytometric analysis of nasal mucosa mMSCs, iPSCs, and iP-MSCs for cell surface markers (CD44, CD73, CD90, and CD105). Data are shown as overlay histograms of the isotype control (light gray) and cell surface marker staining (dark gray). (**2**) RT-PCR analysis of mMSCs, iPSCs, and iP-MSCs of endogenous expression of *SOX2, OCT4, NANOG, LIN28, GDF3*, and *FOXD3*. All iPSCs show high expression of pluripotency-related genes, whereas the expression of these genes was substantially decreased in their parental mMSCs and differentiated iP-MSCs. *GAPDH* was used as housekeeping gene. Mouse embryonic fibroblasts (MEFs) were used as negative control. (**3**) Immunocytochemical analyses of α-SMA and OCT4 (nuclei were stained with DAPI) and the immunohistochemical staining of Vimentin of mMSCs, iPSCs, iP-MSCs (scale bars 100 µm).

Next, we assessed any differences and similarities between originally isolated mMSCs, reprogrammed iPSCs, and differentiated iP-MSCs. An analysis of the expression of typical MSC cell surface markers (CD44, CD73, CD90, CD105) showed that mMSCs and iP-MSCs are nearly identical regarding this expression profile. Interestingly, iPSCs also expressed CD90, whereas they were negative for CD44, CD73, and CD105 (Figure 5 (1)). A comparison of the three cell types mMSCs, iPSCs, and iP-MSCs regarding the expression of pluripotency markers on mRNA level demonstrated that the two independent iPSC lines, which were generated per patient, were highly positive for the pluripotency markers SOX2, OCT4, NANOG, LIN28, GDF3, and FOXD3, whereas mMSCs and iP-MSCs mostly lacked the expression of these genes. Of note, the mMSC lines 2 and 3 as well as the iP-MSC lines 3 and 7 expressed some pluripotency genes at a lower level (Figure 5 (2)). Furthermore, we compared the three cell types regarding protein expression of the mesodermal maker α-SMA, as well as the pluripotency marker OCT4 by immunofluorescence staining. mMSCs, and iP-MSCs expressed α-SMA. Interestingly, undifferentiated iPSCs were also positive for this marker. Unexpectedly, we observed OCT4 in the nucleolus of mMSCs and iP-MSCs, whereas iPSCs were OCT4-positive in the whole nucleus (Figure 5 (3)). The vimentin staining illustrated perfectly the similarities and differences of the investigated stem cells: both mesenchymal stem cell types were highly and consistently positive for vimentin. In contrast, iPSCs were mostly negative for this type 3-intermediary filament (Figure 5 (3)).

### 3.5. IP-MSCs Show Similar Immunosuppressive Capacity as mMSCs

**Figure 6:**
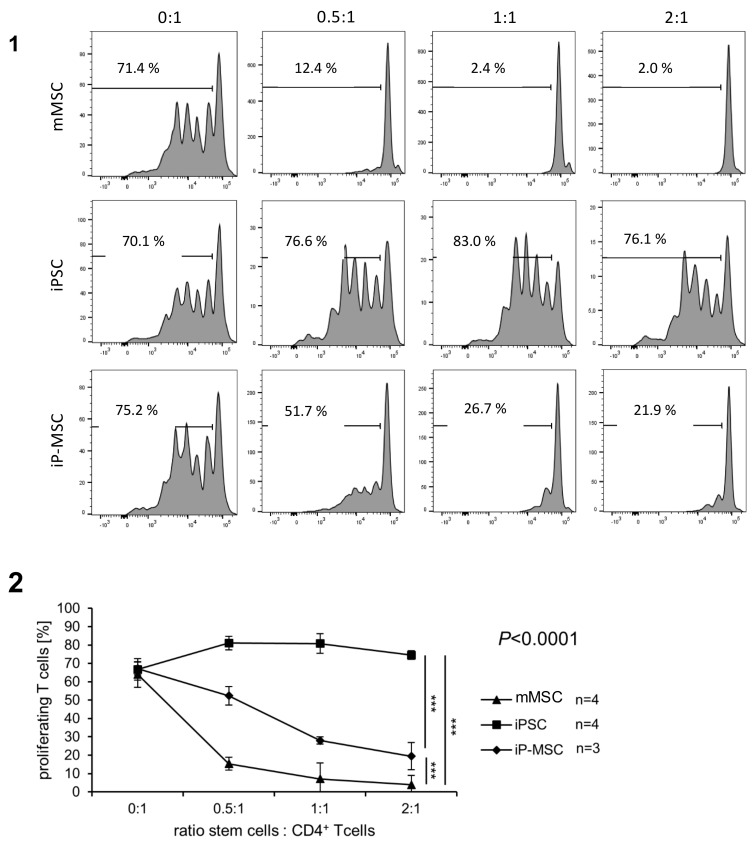
The capacity of nasal mucosa mesenchymal stem cells (mMSCs) to inhibit the proliferation of CD4^+^ T cells is mostly restored in induced pluripotent stem cell-derived mesenchymal stem cells (iP-MSCs) lines. (**1**) The proliferation of carboxyfluorescein succinimidyl ester (CFSE)-stained CD4^+^ T cells was induced by stimulation with anti-CD3 and anti-CD28 antibodies and assayed after 4 days by flow cytometry. The CD4^+^ T cells were cultured alone (0:1) or in the presence of mMSCs, induced pluripotent stem cells (iPSCs), or iP-MSCs derived from patient 7 at various ratios (0.5:1, 1:1, and 2:1). Histograms showing the dilution of CFSE by proliferation are displayed after gating on CD4^+^ T cells. The proportion of proliferating cells in this individual experiment is indicated and was determined using the marker shown in the histograms. (**2**) A summary of results showing means and standard deviations (SD) obtained with several cell lines is displayed. Significant differences between the cell types were revealed by 2-way ANOVA adjusted to the cell ratios 0.5:1, 1:1, and 2:1, and the respective *p*-value is displayed in the diagram. Post hoc analyzes indicated that the inhibitory capacity was different between the cell types (*** *p* < 0.001).

**Figure 7:**
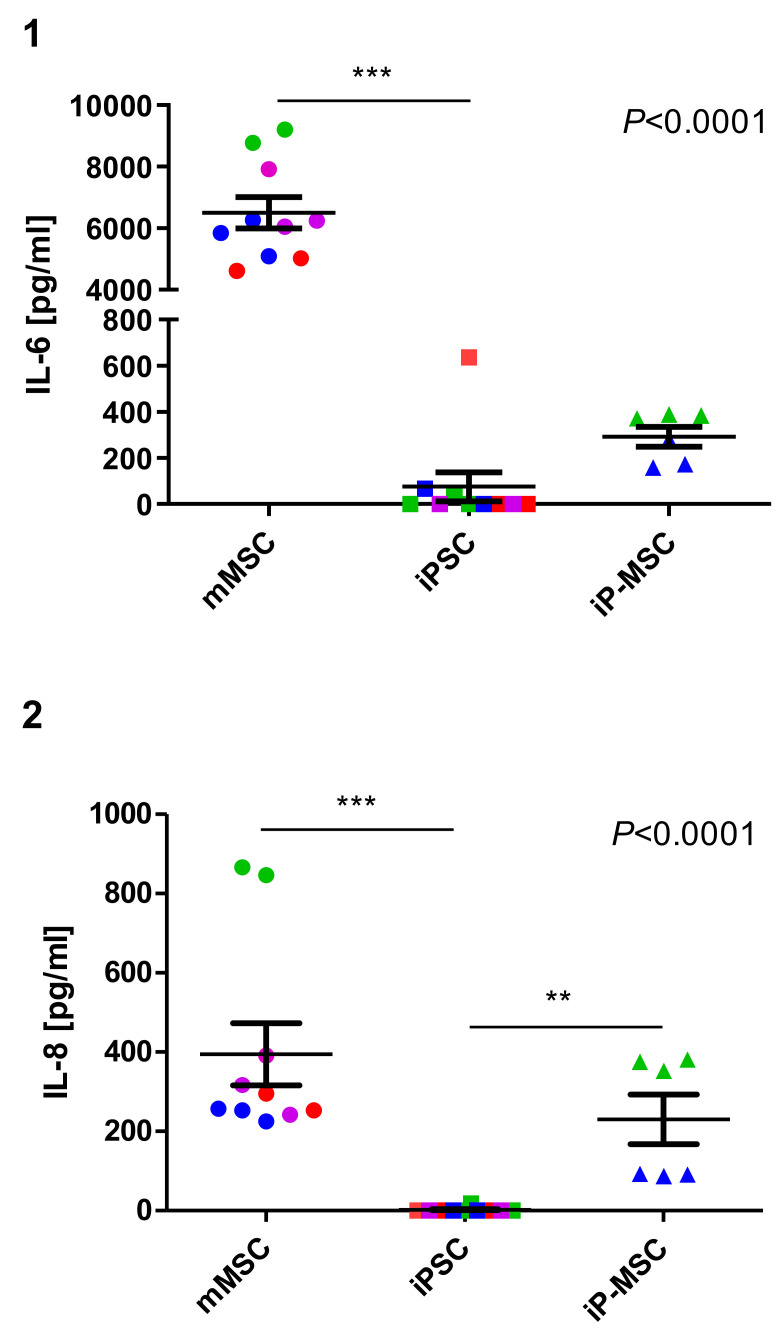
The cytokine profile of nasal mucosa mesenchymal stem cells (mMSCs) is partly restored in induced pluripotent stem-cell-derived mesenchymal stem cells (iP-MSCs) lines. The presence of (**1**) IL-6 and (**2**) IL-8 in the supernatant of MSCs, induced pluripotent stem cells (iPSCs), and iP-MSC after a culture of 24 h was measured by ELISA using cell lines of 4 different patients, which are indicated by color code. Means and standard error of the mean (SEM) are displayed in addition to individual data points. Differences between the cell types were analyzed by Kruskal–Wallis tests, and the respective *p*-values are displayed in the diagrams. Comparisons between individual lines were done by Dunn’s post hoc test, and significant differences are indicated (*** *p* < 0.001, ** *p* < 0.01).

A hallmark of MSCs is their immunosuppressive capacity. Therefore, we assessed the ability of mMSCs, iPSCs, and iP-MSCs to suppress the proliferation of CD4^+^ T cells in response to antigen-specific and co-stimulatory signals delivered by anti-CD3 and anti-CD28 antibodies. As expected, mMSCs were able to completely abolish the proliferation of CD4^+^ T cells. Their reprogramming into iPSCs led to the total loss of this capability, but differentiation of iPSCs into iP-MSCs largely restored this function (Figure 6 (1, 2)). In terms of a patient-specific analysis of inhibition of T cell proliferation, mMSCs from all patients started from a similar level (e.g., T cell:mMSC ratio of 2:1 mMSC7: 1.97%; mMSC5: 2.45%; mMSC2: 4.3%; mMSC3: 7.05%). In contrast, iP-MSCs showed slightly more variability: MSCs of patient 3 showed the highest T cell proliferation inhibition with 13.5%, followed by iP-MSCs of patient 7 (21.9%) and patient 2 (22.9%) (Figure S1 (1, 2)). However, iP-MSCs were less potent than mMSCs, since higher stem cell:CD4+ T cell ratios were required to inhibit the T cell proliferation almost completely. Therefore, we determined the ability of iP-MSCs to produce the cytokines IL-6 and IL-8 in order to gain further insights into their immunological profile. Both cytokines were released by mMSCs in contrast to iPSC as determined in cell culture supernatants by ELISA (Figure 7 (1, 2)). While the secretion of IL-8 was restored in iP-MSCs (Figure 7 (2)), they failed to produce similar amounts of IL-6 as mMSCs (Figure 7 (1), Figure S1 (1)). Notably, mMSCs from patient 3 produced particularly large amounts of IL-8 compared to mMSCs of all other patients, and this feature reoccurred in iP-MSCs since the cells of patient 3 produced more IL-8 than iP-MSCs from patient 7 (Figure 7 (2), Figure S1 (1)). This might be suggestive for a patient-specific immunomodulatory capacity in mMSCs and iP-MSCs, respectively. Conclusions regarding a sex- or age-dependent factor cannot be drawn due to the small sample size. Thus, although iP-MSCs did not show the complete immunological profile of mMSCs with regard to cytokine release, they reacquired major functional properties.

## 4. Discussion

In the present study, we show that 1. MSCs, which exhibit the typical cellular and functional characteristics of tissue-derived MSCs, can be isolated from nasal mucosa; 2. those mMSCs can be efficiently reprogrammed into iPSCs; 3. that MSC-derived iPSCs can be reprogrammed into functional iP-MSCs (Figure 1). We were able to show that iP-MSCs showed nearly identical cell surface marker expression to the original mMSCs and that iP-MSCs reacquired major functional properties. Previous reports have demonstrated either the isolation and characterization of MSCs or the differentiation of iPSCs into iP-MSCs. The focus of our study was the combination of the described methods (isolation, reprogramming, and differentiation), resulting in three cell types, all derived from the same human origin. We aimed to investigate similarities and differences in both cell characteristics and immunomodulatory capacity of primary isolated mMSCs and generated autologous iPSC-derived MSCs. This opens the possibility to use the generated iP-MSCs in regenerative medicine in an autologous manner.

Following the findings of several other studies in which functional MSCs were isolated from various tissues like bone-marrow and fat, as well as from the head and neck region, we have isolated MSCs from nasal mucosa [21,33,34]. Our mMSCs show an intermediate stage of potency with some pluripotency marker genes being expressed, as shown in Figure 5 (2). This is in line with a report from Pierantozzi et al. that MSCs from bone marrow and fat tissue are positive for OCT4 and NANOG [35]. Like others [36,37,38], we have shown that isolated and generated mMSCs express cell surface markers such as CD44, CD73, and CD105, whereas iPSCs are negative for these antigens but positive for CD90, showing that CD90 is an unsuitable marker for defining a mesenchymal phenotype.

With respect to MSC-derived iPSCs, we were able to induce the expression of endogenous pluripotency markers like OCT4, NANOG, LIN28, and SOX2 on mRNA and protein level, which were mostly absent in the multipotent cell types (both mMSCs and iP-MSCs). These markers are essential for the maintenance of pluripotency, self-renewal capacity, and prevention of differentiation [39,40]. However, low mRNA levels of the pluripotency marker genes NANOG and LIN28 were observed in patient 3 mMSCs, the youngest patient at the age of 21, suggesting that some mMSCs exhibit elements of the phenotype of pluripotent cells. This is in line with previous studies demonstrating pluripotency marker expression for MSCs from bone marrow and fat tissue [35].

We have shown the differentiation of iPSCs into iP-MSCs as described previously [37,41] and were able to generate iP-MSCs fulfilling all criteria established by the International Society of Cellular Therapy for defining an MSC population [42]. Our iP-MSCs were capable of trilineage differentiation, in contrast to previous studies [43], and although they did not show the complete immunological profile of mMSCs with regard to cytokine release, they reacquired major functional properties like suppression of CD4^+^ T cell proliferation. The decreased cytokine release of iP-MSCs compared to mMSCs might be due to a higher amount of non-MSC-like cells or not-mature cells among the iP-MSC than among the MSC populations.

In the literature, it is controversially discussed whether iPSCs have an epigenetic memory of their origin of tissue, thereby exhibiting an increased propensity for differentiation into the original tissue [38,44]. Bar-Nur and colleagues were able to show that iPSCs reprogrammed from pancreatic beta cells had a higher ability to differentiate into insulin-producing cells in comparison to non-beta-cell-derived iPSCs [45]. Here, we demonstrated that mMSCs from patient 3 produced large amounts of IL-6 and IL-8 compared to the other three patients. Since iP-MSCs from patient 3 produced also more IL-6 and IL-8 than iP-MSCs from patient 7, this feature might be the result of iP-MSCs’ epigenetic memory of the original mMSCs. Thus, epigenetic memory can be exploited to acquire specific cells from iPSCs. These findings indicate that iP-MSCs hold great promise in patient-individualized medicine due to their similarities to isolated MSCs. Moreover, iP-MSCs are suitable for further immunology experiments, drug screening, and tissue regeneration [46].

Radiotherapy is often an element of a multimodality approach for the curative treatment of head and neck squamous cell carcinoma. It is accompanied by adverse effects resulting from damage of healthy tissue surrounding the treated area [1]. Mucositis, pain, and xerostomia result in short- and long-term side effects of radiation therapy, which significantly compromise the patient’s quality of life [47,48]. These side effects can be minimized by enhanced tissue regeneration of the affected area, which is largely dependent on the survival and regenerative capabilities of tissue-resident or invading stem cells [3,4]. Tissue-resident MSCs open the opportunity of patient-individualized treatment due to their regenerative and immunological abilities. The isolation of stem cells from patient tissue has been described thoroughly in the past [49]. However, in vitro growth and multiplying of MSC subside at some point, which leads to a significant disadvantage in clinical and practical application [50,51]. To overcome this hurdle, iPSCs reprogrammed from several somatic original materials can be generated as cell types with self-renewal capacity. Currently, the isolation of mMSCs prior to the beginning of radiation therapy is debated [52,53]. Those mMSCs could be reprogrammed in iPSCs, differentiated in autologous iP-MSCs, and then given to patients during or after therapy. Concerning adipogenic stem cells, in vivo experiments in a murine model were first performed with isolated adipogenic stem cells and iP-MSCs [54]. The benefit of this personalized treatment would be the autologous transplantation, which lacks the risk of rejection reaction. Simultaneously, the patient could benefit from immunomodulatory properties of the cells.

It is known that IL-6 regulates angiogenesis, collagen accumulation, and leucocyte infiltration, and in addition is involved in cell proliferation, apoptosis, survival, and differentiation [55,56]. IL-8 stimulates the deposition of fibronectin and collagen during the process of wound healing and promotes cell migration and chemotaxis of keratinocytes [57,58,59]. Both mMSCs and iP-MSCs in our study secreted IL-6 and IL-8, which underlines the immunomodulatory capacities of iP-MSCs.

One key challenge to our new method is its reproducibility, standardization, and efficiency. The isolation of MSCs from the nasal mucosa is a simple and straightforward process. We managed to isolate the cells and reprogram and differentiate them to iP-MSCs within 16 to 18 weeks. Furthermore, the iPSCs are suitable as an endless stack for patients’ cells and material to differentiate more specific cells for the patients need.

## 5. Conclusions

In the present study, we generated induced pluripotent stem cells from isolated human mMSCs of the upper respiratory tract and differentiated them back into corresponding induced pluripotent stem cell-derived MSCs. Both mesenchymal stem cell types were highly similar in terms of morphology, clonogenic and differentiation potential, and cell surface marker expression. They also shared an immunomodulatory potential with respect to the secretion of cytokines and the ability to inhibit T cell proliferation. Therefore, they might hold great potential in numerous clinical applications.

## Figures and Tables

**Table 1:** Age and sex of the patients.

**Patient**	mMSC2	mMSC3	mMSC5	mMSC7
**Age**	41	21	39	77
**Sex**	female	male	female	male

## Data Availability

The data presented in this study are available in the article and supplementary material here.

## Supplementary Materials

The following are available online at https://www.mdpi.com/2073-4409/10/1/33/s1, Table S1: Antibodies used in Fluorescence-activated cell sorting, Table S2: Antibodies used in Immunocytochemistry, Table S3: Primers used in RT-PCR, Table S4: Cell surface marker expression on a patient-specific level; Figure S1: Immunomodulatory potential of nasal mucosa mesenchymal stem cells (mMSCs), induced pluripotent stem cells (iPSC) and induced pluripotent stem cell-derived mesenchymal stem cells (iP-MSCs) on a patient-specific level.
