# Supplementary material for: Pluripotent Stem Cell-Derived Mesenchymal Stem Cells Show Comparable Functionality to Their Autologous Origin
Source: Cells. 2020 Dec 28;10(1):33. doi: 10.3390/cells10010033 (PMC7823915; doi:10.3390/cells10010033)

Supplemental Table S1: Antibodies used in Fluorescence activated cell sorting

Antibody	Clone	Dilution	Supplier
Pacific Blue anti-human CD11b	ICRF44	0.2 mg/ml	BioLegend, San Diego, USA
PE anti-human CD34	561	25 µg/ml	BioLegend, San Diego, USA
FITC anti-human CD44	BJ18	200 µg/ml	BioLegend, San Diego, USA
Pacific Blue anti-human CD45	H130	0.5 mg/ml	BioLegend, San Diego, USA
APC anti-human CD73	AD2	100 µg/ml	BioLegend, San Diego, USA
FITC anti-human CD90 (Thy1)	5E10	200 µg/ml	BioLegend, San Diego, USA
APC anti-human CD105	43A3	100 µg/ml	BioLegend, San Diego, USA
**Secondary antibody**	**Type**	**Dilution**	**Supplier**
APC Mouse IgG1, k isotpye Ctrl (FC)	MOPC-21	200 µg/ml	BioLegend, San Diego, USA
Pacific Blue Mouse IgG1 k isotype Ctrl	MOPC-21	0.5 mg/ml	BioLegend, San Diego, USA
PE Mouse IgG2a, k isotype Ctrl	MOPC-173	0.2 mg/ml	BioLegend, San Diego, USA
FITC Mouse IgG1, k isoype Ctrl	MOPC-21	2,5 mg/µl	BioLegend, San Diego, USA

Supplemental Table S2: Antibodies used in Immunocytochemistry

Primary antibody	Type	Dilution in 1% BSA	Supplier
NANOG	Polyclonal goat IgG	1:200 (+0.1% Triton-X)	R&D Systems, Minneapolis, USA
OCT4	Polyclonal goat IgG	1:40 (+0.1% Triton-X)	R&D Systems, Minneapolis, USA
SOX2	monoclonal mouse IgG2A	1:50 (+0.1% Triton-X)	R&D Systems, Minneapolis, USA
LIN28	Polyclonal goat IgG	1:300 (+0.1% Triton-X)	R&D Systems, Minneapolis, USA
TRA1-60	Monoclonal mouse IgM	1:200	Abcam, Cambridge, UK
SSEA4	monoclonal mouse IgG2A	1:200	Abcam, Cambridge, UK
AFP	Polyclonal rabbit IgG	1:100 (+0.1% Triton-X)	Dako, Hamburg, Germany
α-SMA	Monoclonal mouse IgG2A	1:3000 (+0.1% Triton-X)	Sigma Aldrich, St. Louis,USA
βIII-tubulin	mouse IgG2A	1:1000 (+0.1% Triton-X)	BioLegend, San Diego, USA
**Secondary antibody**	**Type**	**Dilution**	**Supplier**
Alexa Fluor 555 α-goat	Donkey-α-goat IgG	1:1000	Thermo Fischer Scientific, Waltham USA
Alexa Fluor 488 α-mouse	Donkey-α-mouse IgG	1:1000	Thermo Fischer Scientific, Waltham USA
Alexa Fluor 555 α-rabbit	Donkey-α-goat IgG	1:1000	Thermo Fischer Scientific, Waltham USA
Cyanine Cy3 α-mouse	Goat-α-mouse IgG+IgM	1:300	Jackson Immuno Research, West Grove, USA

Supplemental Table S3: Primers used in RT-PCR

Primer	Forward primer sequence	Reverse primer sequence	Product size (bp)	Annealing temperature	Cycle
GAPDH	AGAGGCAGGGATGATGTTCT	TCTGCTGATGCCCCCATGTT	258	58°	30
SOX2	ATGCACCGCTACGACGTGA	CTTTTGCACCCCTCCCATTT	437	58°	30
GDF3	TTCGCTTTCTCCCAGACCAAGGTTTC	TACATCCAGCAGGTTGAAGTGAACAGCACC	311	58°	34
OCT4	GACAACAATGAAAATCTTCAGGAGA	TTCTGGCGCCGGTTACAGAACCA	218	58°	32
FOXD3	GTGAAGCCGCCTTACTCGTAC	CCGAAGCTCTGCATCATGAG	353	58°	38
LIN28	AGTAAGCTGCACATGGAAGG	ATTGTGGCTCAATTCTGTGC	410	58°	36
NANOG	AGTCCCAAAGGCAAACAACCCACTTC	ATCTGCTGGAGGCTGAGGTATTTCTGTCTC	164	64°	30
AFP	ACTCCAGTAAACCCTGGTGTTG	GAAATCTGCAATGACAGCCTCA	255	58°	30
CTNT	GACAGAGCGGAAAAGTGGGA	TGAAGGAGGCCAGGCTCTAT	305	58°	30
TH	GCGGTTCATTGGGCGCAGG	CAAACACCTTCACAGCTCG	215	60°	38

Supplemental Table S4: Cell surface marker expression of nasal mucosa mesenchymal stem cells (mMSCs), induced pluripotent stem cells (iPSC) and induced pluripotent stem cell-derived mesenchymal stem cells (iP-MSCs) on a patient-specific level.

cell line	CD11b	CD34	CD44	CD45	CD73	CD90	CD105
mMSC2	70	0	20782	31	29149	24069	862
iP-MSC2	630	0	87803	85	115375	18151	3106
mMSC3	20	0	20615	86	23350	7362	271
iP-MSC3	0	0	44054	0	58954	24548	3971
mMSC5	21	22	15743	27	26568	23388	685
iP-MSC5	n. a.	n. a.	n. a.	n. a.	n. a.	n. a.	n. a.
mMSC7	15	22	18377	33	28433	28265	605
iP-MSC7	89	0	9758	45	8855	4182	350

Supplemental Figure S1: Immunomodulatory potential of nasal mucosa mesenchymal stem cells (mMSCs), induced pluripotent stem cells (iPSC) and induced pluripotent stem cell-derived mesenchymal stem cells (iP-MSCs) on a patient-specific level. 1 Inhibition of $CD4^+$ T cell proliferation at different $CD4^+$:MSC/iPSC ratios (0:1; 0.2:1, 1:1, and 2:1) and the presence of IL-6 and IL-8 in the supernatant of mMSC, iPSC, and iP-MSC after a culture of 24 hours measured by ELISA using cell lines of 4 different patients. **2** Individual inhibition of $CD4^+$ T cell proliferation of mMSC, iPSC, and iP-MSC from each patient.

1

ratio CD4:MSC/iPSC	0:1	0,5:1	1:1	2:1	**IL-6 [pg/ml]**	**IL-8 [pg/ml]**
**cell line**	proliferating T cells [%]				average [SD]	average [SD]
mMSC2	65.4	11.9	4.75	4.3	4817.3 [286.2]	274.5 [30.1]
iPSC2	64.7	88.1	81.5	63.7	106.1 [183.8]	0
iP-MSC2	63.3	56.2	37.3	22.9	n.a.	n.a.
mMSC3	57.4	18.1	6.6	7.05	8991.3 [304.1]	866.4 [0]
iPSC3	62.8	78.4	80.6	79.6	7.9 [13.8]	3.1 [5.4]
iP-MSC3	63	49.2	20.1	13.5	381.5 [9.2]	370.1 [15.3]
mMSC5	61.8	19	14.7	2.45	5732.7 [602.2]	244.8 [17.3]
iPSC5	69.2	81.4	78.2	78.8	16.7 [23.6]	0
iP-MSC5	n.a.	n.a.	n.a.	n.a.	n.a.	n.a.
mMSC7	71.4	12.4	2.43	1.97	6736.0 [1027.7]	316.7 [74.4]
iPSC7	70.1	76.6	83	76.1	0	0
iP-MSC7	75.2	51.7	26.7	21.9	202.7 [65.9]	90.6 [2.5]

2

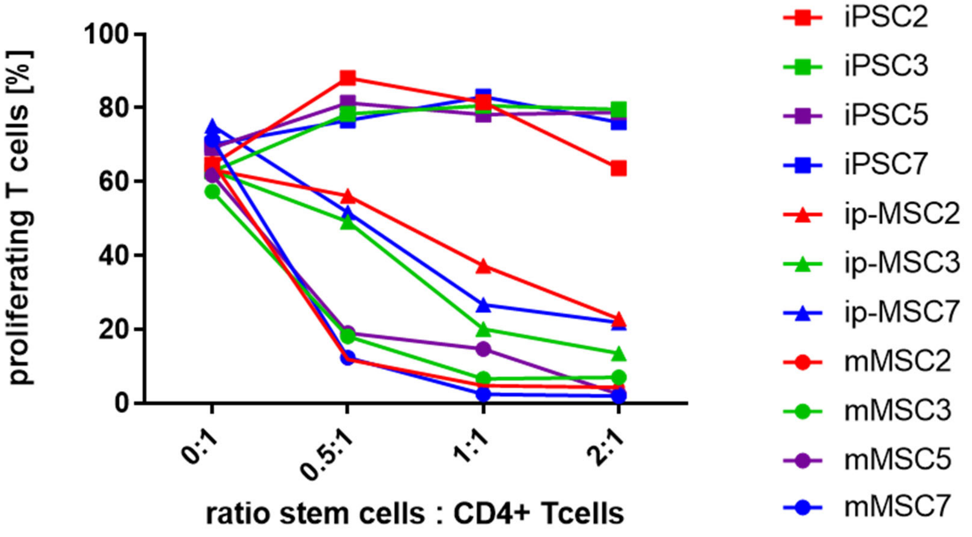

Supplement: Supplementary file 1 [file cells-10-00033-s001.pdf]
